# Wetland-to-Meadow Transition Alters Soil Microbial Networks and Stability in the Sanjiangyuan Region

**DOI:** 10.3390/microorganisms13061263

**Published:** 2025-05-29

**Authors:** Guiling Wu, Jay Gao, Zhaoqi Wang, Yangong Du

**Affiliations:** 1 State Key Laboratory of Plateau Ecology and Agriculture, Qinghai University, Xining 810016, China; wangzhaoqi@qhu.edu.cn; 2 School of Environment, University of Auckland, P.O. Box 92019, Auckland 1142, New Zealand; jg.gao@auckland.ac.nz; 3 Qinghai Provincial Key Laboratory of Restoration Ecology for Cold Region, Northwest Institute of Plateau Biology, Chinese Academy of Sciences, Xining 810008, China; ygdu@nwipb.cas.cn

**Keywords:** wetland degradation, microbial co-occurrence networks, functional thresholds, ecological restoration, Qinghai–Tibet Plateau

## Abstract

Wetlands and meadows are two terrestrial ecosystems that are strikingly distinct in terms of hydrological conditions and biogeochemical characteristics. Wetlands generally feature saturated soils, high accumulation of organic matter, and hypoxic environments. They support unique microbial communities and play crucial roles as carbon sinks and nutrient retainers. In contrast, meadows are characterized by lower water supply, enhanced aeration, and accelerated turnover of organic matter. The transition from wetlands to meadows under global climate change and human activities has triggered severe ecological consequences in the Sanjiangyuan region, yet the mechanisms driving microbial network stability remain unclear. This study integrates microbial sequencing, soil physicochemical analyses, and structural equation modeling (SEM) to reveal systematic changes in microbial communities during wetland degradation. Key findings indicate: (1) critical soil parameter shifts (moisture: 48.5%→19.3%; SOM: −43.6%; salinity: +170%); (2) functional microbial restructuring with drought-tolerant Actinobacteria (+62.8%) and Ascomycota (+48.3%) replacing wetland specialists (Nitrospirota: −43.2%, Basidiomycota: −28.6%); (3) fundamental network reorganization from sparse wetland connections to hypercomplex meadow networks (bacterial nodes +344%, fungal edges +139.2%); (4) SEM identifies moisture (λ = 0.82), organic matter (λ = 0.68), and salinity (λ = −0.53) as primary drivers. Particularly, the collapse of methane-oxidizing archaea (−100%) and emergence of pathogenic fungi (+28.6%) highlight functional thresholds in degradation processes. These findings provide microbial regulation targets for wetland restoration, emphasizing hydrologic management and organic carbon conservation as priority interventions. Future research should assess whether similar microbial and network transitions occur in degraded wetlands across other alpine and temperate regions, to validate the broader applicability of these ecological thresholds. Restoration efforts should prioritize re-saturating soils, reducing salinity, and enhancing organic matter retention to stabilize microbial networks and restore essential ecosystem functions.

## 1. Introduction

Wetland ecosystems, one of the most productive ecosystem types on the Earth’s surface, possess irreplaceable ecological functions in maintaining biodiversity [1], regulating regional hydrological cycles [2], and storing organic carbon in terrestrial ecosystems [3]. However, under the dual pressures of intensified global climate change and human activities, more than 35% of natural wetlands globally are undergoing degradation to varying degrees [4]. This degradation process is particularly pronounced in the alpine wetland ecosystems of the Sanjiangyuan region in China. The latest monitoring data show that the wetland area in this region has decreased by 28.6% over the past 30 years, with a degradation rate 2.3 times that of regions at the same latitude [5]. The resulting decline in ecosystem functions has posed a potential threat to the ecological security of hundreds of millions of people downstream [6].

Current studies have revealed the main driving mechanisms of wetland degradation. The thawing of permafrost caused by climate change has led to an annual average decline in the groundwater level of 12–18 cm [7], while human disturbances, such as overgrazing, have accelerated the degradation process of vegetation [8]. These environmental changes affect the structure and function of microbial communities by altering soil physical and chemical properties [9] and vegetation composition [10]. Existing research indicates that wetland desiccation can cause a 43–51% decrease in soil organic matter content [11], a shift of dominant microbial groups from anaerobic to drought-tolerant types [12], and a significant reduction in the abundance of key functional genes for carbon and nitrogen cycling [13].

Nevertheless, there are still some crucial scientific gaps in current research. For instance, most studies focus on single-dimensional analyses of plant communities and soil physical and chemical indicators, lacking a systematic analysis of the coupling relationship among microbial community structure–function–environmental factors [14,15,16,17]. There is insufficient understanding of the dynamic response mechanisms of the stability of microbial ecological networks during the wetland–grassland transformation process [18]. Moreover, a driving model of multi-environmental gradient factors in the succession of microbial communities has not been established [19].

In this study, for the typical wetland–grassland transformation sequence in the Sanjiangyuan region, an integrated analysis framework is constructed for the first time, which includes multi-dimensional microbial characteristics (community composition, niche breadth, co-occurrence network), key soil factors (moisture, organic matter, salinity), and environmental gradients (freeze–thaw characteristics, vegetation characteristics). The limitations of existing research are addressed by solving the following scientific questions: (1) How do microbial diversity, functional genes, and ecological network topological structures respond during the wetland desiccation process? (2) How do changes in soil moisture dynamics and nutrient availability drive the restructuring of microbial communities? (3) Which key environmental factors dominate the succession of microbial communities during the wetland–grassland transformation process?

Based on the above questions, the following research hypotheses are proposed:

**H1.**  *Wetland degradation reduces microbial diversity and destabilizes ecological network structure, with particularly strong impacts on functional groups involved in carbon and nitrogen cycling.*

**H2.**  *Soil moisture and organic matter are the key mediators driving the shift in microbial communities from anaerobic to drought-tolerant types.*

**H3.**  *Microbial community succession is jointly influenced by soil moisture, salinity, and vegetation structure, with soil moisture playing the most dominant role.*

The implementation of this study will reveal, for the first time, the adaptive regulation mechanism of microbial communities during the degradation of alpine wetlands and establish an interaction model for soil–vegetation–microorganisms. The research results can provide scientific support for wetland restoration projects in Sanjiangyuan National Park in two aspects: identifying early warning indicators of microbial community degradation and formulating ecological restoration strategies based on the regulation of key driving factors. This has important practical value for maintaining the ecological barrier function of the Qinghai–Tibet Plateau and achieving regional sustainable development.

## 2. Materials and Methods

### 2.1. Study Area

**Figure 1:**
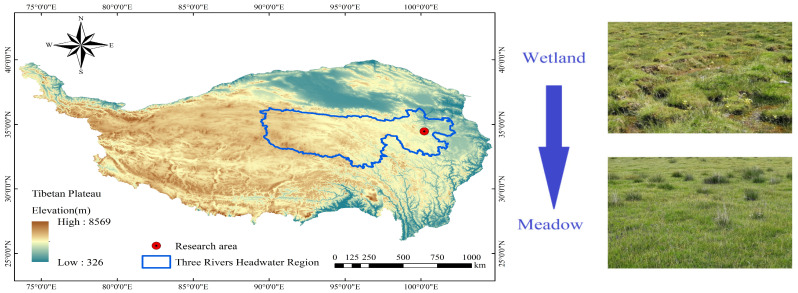
Spatial distribution of sampling sites of typical wetland regions (with hummocks) and the appearance of meadow degraded from wetland. Northeastern Qinghai–Tibet Plateau, China.

This study was conducted in the alpine wetland of Dawutan, located in Maqin County, Golog Prefecture, Qinghai Province, within the northeastern part of the Qinghai–Tibet Plateau (Sanjiangyuan region). The core research site is situated at approximately 34°27′ N, 100°12′ E, with an average elevation of 3688 m above sea level (Figure 1). The area exhibits typical plateau climatic characteristics: long, cold winters and short, cool summers. The mean annual temperature is –2.6 °C, and average annual precipitation is 513 mm, concentrated mainly during the growing season from May to September. There is no true frost-free period, and the region receives about 2576 h of sunshine annually [2,20]. This area was selected as representing a typical case of wetland-to-meadow degradation driven by both climate change and human activities. Since the 1980s, overgrazing and rising temperatures have led to groundwater depletion, vegetation shifts, and declines in ecosystem function [1,10,21].

A 340-hectare degradation gradient zone was selected, encompassing the natural transition from alpine wetland to alpine meadow. Ten sampling plots were established along this gradient—five in the wetland area and five in the meadow area. Each plot measured 10 m × 10 m and was spaced at least 1000 m apart to reduce spatial autocorrelation. Within each plot, six 1 m × 1 m quadrats were randomly placed for vegetation surveys, and soil samples were collected simultaneously. All plot locations were recorded using a handheld GPS with ±3 m accuracy. The two habitat types surveyed include the following: (a) wetland plots (W1–W5): located in the saturated, peat-rich core of the alpine marsh wetland, dominated by *Kobresia tibetica*, at approximately 34°27′15″ N, 100°11′45″ E; (b) meadow plots (M1–M5): situated approximately 5 km from the wetland core, in a well-drained alpine meadow dominated by *Kobresia humilis*, at approximately 34°26′50″ N, 100°12′55″ E.

### 2.2. Sample Collection

A total of 60 sampling quadrats were established in the field, including 30 in wetland areas and 30 in meadow areas, to conduct integrated vegetation and soil surveys. For vegetation sampling, the dominant plant species, vegetation cover, species richness, and diversity indices were recorded in each quadrat. Aboveground biomass (AGB) was collected using a 0.25 m^2^ harvesting frame, with three replicates per quadrat. All samples were dried in the laboratory at 80 °C to a constant weight. Belowground biomass (BGB) was sampled using a root auger with a diameter of 5 cm and a depth of 20 cm. To avoid mixing errors, samples were divided into two layers: 0–10 cm and 10–20 cm [5,6]. All plant samples were sealed in plastic bags, transported to the laboratory, and oven-dried at 80 °C until constant weight was achieved. For soil sampling, five evenly distributed points were selected within each quadrat, and 0–20 cm deep soil was collected using a hand auger. The samples were mixed into a single composite sample per quadrat, resulting in a total of 60 soil samples.

Each soil sample was divided into three portions according to analytical purposes. (1) Microbial DNA analysis: Approximately 10 g of fresh soil was manually homogenized, with visible plant residues and stones removed. The sample was immediately snap-frozen in liquid nitrogen (–196 °C for 5 min) in the field, sealed in sterile cryovials, and transported to the laboratory on dry ice. Samples were stored at –80 °C for subsequent DNA extraction and high-throughput sequencing to preserve enzymatic and DNA integrity as much as possible. (2) Soil physicochemical analysis: A portion of the soil was sieved through a 2 mm mesh to remove debris and stones, then air-dried at room temperature. These samples were used to determine soil organic matter, moisture content, pH, salinity, and other physicochemical properties. (3) Microbial activity analysis: Approximately 100 g of intact moist soil was sealed in a plastic bag and stored at 4 °C for microbial respiration and enzyme activity measurements. In the wetland microtopography survey, measurements were conducted only in wetland plots. A stainless steel ruler was used to record the aboveground water film depth (AWFD) and the height of hummocks (AWFH). In total, 270 hummocks were measured for height and diameter, and average values were calculated for use in microtopographic structure analysis.

### 2.3. Data Analysis

(1) Bacterial and Fungal Community Composition and Niche Breadth Indices

The composition and abundance of bacterial (16S rRNA) and fungal (ITS) communities, as well as the niche breadth indices, were determined through the following steps. (a) Soil Sample Preparation: Soil samples were stored at −80 °C for subsequent DNA extraction. Plant roots, stones, and other impurities were removed, and the soil samples were homogenized using a homogenizer to ensure their representativeness [22]. (b) DNA Extraction: Soil DNA was extracted using commercial soil DNA extraction kits (e.g., Qiagen DNeasy PowerSoil Kit, manufactured by Qiagen Inc., Hilden, Germany), following the manufacturer’s protocol [23]. Special attention was paid to removing inhibitors (e.g., humic acids) to avoid interference in downstream analyses. The concentration and purity of the extracted DNA were quantified using a NanoDrop, ensuring that the 260:280 ratio was between 1.8 and 2.0 [24]. DNA Extraction Quality Control was performed using the QIAGEN DNeasy PowerSoil Pro Kit (optimized for soils with high humic acid content), and a negative control (sterile water) was included in each batch. DNA Purity Verification was performed using the A260/A280 ratio, which was required to be between 1.8 and 2.0, and the A260/A230 ratio, which had to be >1.5 (detected by NanoDrop). Samples that did not meet these standards were re-extracted. (c) Polymerase Chain Reaction (PCR) Amplification: The V3-V4 hypervariable region of the bacterial 16S rRNA gene was amplified using primers 338F (5′-ACTCCTACGGGAGGCAGCAG-3′) and 806R (5′-GGACTACHVGGGTWTCTAAT-3′) [25]. For the fungal ITS1 or ITS2 regions, the commonly used primer pairs ITS1F (5′-CTTGGTCATTTAGAGGAAGTAA-3′) and ITS2 (5′-GCTGCGTTCTTCATCGATGC-3′) were employed [26]. PCR amplification was optimized as follows: for the bacterial 16S rRNA V3-V4 region, primers 338F/806R were used, with single-indexing (Illumina Nextera XT Index Kit, manufactured by Illumina, Inc., San Diego, CA, USA) added to distinguish samples; for the fungal ITS1 region with primers ITS1F/ITS2, a Touchdown PCR program (annealing temperature decreased from 65 °C to 55 °C) was adopted to improve specificity. A positive control (standard strain DNA) and a negative control (no template) were set for each plate. (d) High-Throughput Sequencing: PCR products were purified using Ampure XP magnetic beads to remove primers and non-specific amplification fragments, and the library quality was assessed [27]. Sequencing was performed on the Illumina MiSeq platform (2 × 300 bp paired-end sequencing). Sequencing Data Quality Control involved filtering raw data using Trimmomatic (Phred ≥ 20, length ≥ 150 bp).Denoising was carried out using DADA2 software (version 1.20.0, https://benjjneb.github.io/dada2/, accessed on 15 March 2025) to generate an ASV table, and singleton ASVs were removed. (e) Data Analysis: Sequencing data were processed using QIIME2 (version 2024.2, https://qiime2.org/, accessed on 10 March 2025) and DADA2 (version 1.20.0, https://benjjneb.github.io/dada2/, accessed on 15 March 2025) software for quality control, which included removing low-quality sequences, chimera filtering, and sequence merging [28]. Sequences were clustered into operational taxonomic units (OTUs) based on 97% similarity or processed into amplicon sequence variants (ASVs) using DADA2 [29]. Taxonomic annotation was performed using the SILVA database for bacteria and the UNITE database for fungi [30]. Diversity analyses included calculating alpha diversity indices (e.g., Shannon index, Chao1 index) and beta diversity metrics (e.g., Bray-Curtis distances).The Levins’ niche breadth index was calculated using the “MicroNiche” package (version 1.0.2, https://cran.r-project.org/package=MicroNiche, accessed on 20 February 2025) in R. Intergroup difference tests between communities were conducted using ANOSIM (community structure analysis) and LEfSe (biomarker species identification) in R. Functional predictions were performed using PICRUSt2 to annotate bacterial KEGG pathways, FUNGuild to classify fungal trophic modes, and ASV abundances to estimate the potential expression of functional genes. Statistical Tests: ANOSIM (Analysis of Similarities) was used to compare the overall community composition among groups. The ANOSIM statistic tests the null hypothesis that the groups’ communities are similar, with *p*-values < 0.05 considered significant. LEfSe (Linear discriminant analysis effect size) was used to identify biomarkers that differ significantly between the groups. This method integrates statistical significance with biological relevance. Alpha diversity indices were calculated to estimate microbial diversity within each sample. The Shannon index and Chao1 index provide insights into the species richness and evenness, with higher values indicating greater diversity. Beta diversity was calculated using Bray–Curtis dissimilarities to compare microbial community composition across samples. This method quantifies the dissimilarity between pairwise samples, and clustering was visualized using ordination techniques like PCoA. (f) Co-occurrence Network Construction: To investigate microbial interactions, co-occurrence networks were built based on ASV abundance. Rare ASVs (present in <20% of samples or with relative abundance < 0.01%) were removed. Spearman correlations (|r| > 0.6, *p* < 0.01) were calculated using the “Hmisc” package in R (version 5.1-1, https://cran.r-project.org/package=Hmisc, accessed on 25 February 2025), and significant correlations were used to construct networks in Gephi (v0.9.2). Network metrics, including node number, edge number, modularity, and clustering coefficient, were computed to assess network complexity and stability. Network robustness was tested by simulating random node removal, and key taxa were identified based on centrality and degree.

(2) Soil Physicochemical Properties

Soil organic carbon (TOC) was determined using an elemental analyzer (Elementar Vario EL III), and SOM was calculated using the formula SOM = TOC × 1.724 [31]. Alkaline hydrolysable nitrogen (AN) was measured by the alkaline hydrolysis diffusion method, total nitrogen (TN) by the Kjeldahl method, and soil moisture content using the oven-drying method [32]. Soil salinity (EC) was determined by the saturated extract method (soil:water = 1:5) using a conductivity meter (METTLER TOLEDO), with temperature correction to 25 °C [8]. For dynamic monitoring of soil moisture, soil moisture sensors (Decagon EC-5) were used to continuously record the volumetric water content (VWC, %) at a depth of 0–20 cm, with real-time data collected during sampling.

(3) Environmental Gradient Analysis

The mantel function in the vegan package (version 2.6-4, https://cran.r-project.org/package=vegan, accessed on 28 February 2025) in R was used to assess the relationship between environmental gradients and microbial community similarity [33]. Bray–Curtis distances were calculated to evaluate environmental distances and microbial community similarity [34]. The Mantel test was used to calculate the Spearman correlation coefficient between environmental distance and microbial community similarity, with permutation tests to determine significance [35]. The SparCC algorithm was employed for network construction (with 100 iterations, *p* < 0.01), and weak correlation edges (|r| < 0.6) were filtered. Topological parameters were calculated, including node centrality (Degree centrality, Closeness centrality, and Betweenness centrality) and network robustness. Network stability was tested through random node deletion simulations. Statistical Methodology: The Mantel test was used to assess the relationship between environmental gradients (e.g., moisture, SOM, salinity) and microbial community composition, with a *p*-value < 0.05 considered statistically significant. The lavaan package (version 0.6-15, https://cran.r-project.org/package=lavaan, accessed on 5 March 2025 in R) was used for SEM to assess the influence of soil moisture, SOM, and salinity (as exogenous variables) on microbial diversity (Shannon index) and functional gene abundances (as endogenous variables). Model fitting was performed using maximum likelihood estimation, with criteria CFI > 0.95, RMSEA < 0.05, and χ^2^/df < 2.

## 3. Results

### 3.1. Vegetation and Environmental Variables

In alpine wetlands (hereafter referred to as “wetland”), the plant community is dominated by Kobresia tibetica (Cyperaceae), whereas in alpine meadows (“meadow”), Kobresia pygmaea (Cyperaceae) is the dominant species. The aboveground biomass, Cyperaceae biomass, and belowground biomass in wetlands were significantly higher than those in meadows, while weed biomass was significantly greater in meadows than in wetlands. There were notable differences in abiotic properties between the two ecosystems. Wetland soils exhibited significantly higher levels of soil organic matter (SOM), soil organic carbon (SOC), dissolved organic carbon (DOC), total nitrogen (TN), available potassium (K), carbon-to-nitrogen ratio (C:N), SOC density, soil water content (SWC), and microbial indices compared to meadow soils. In contrast, meadows had significantly higher soil pH, soil bulk density (SBD), electrical conductivity (EC), soil respiration rate, and soil carbon metabolic activity. No significant differences were observed between wetlands and meadows in terms of available phosphorus and the biomass of Poaceae species (Supplementary Table S1).

### 3.2. Characteristics of Soil Microbial Community Composition in Wetlands and Meadows

**Figure 2:**
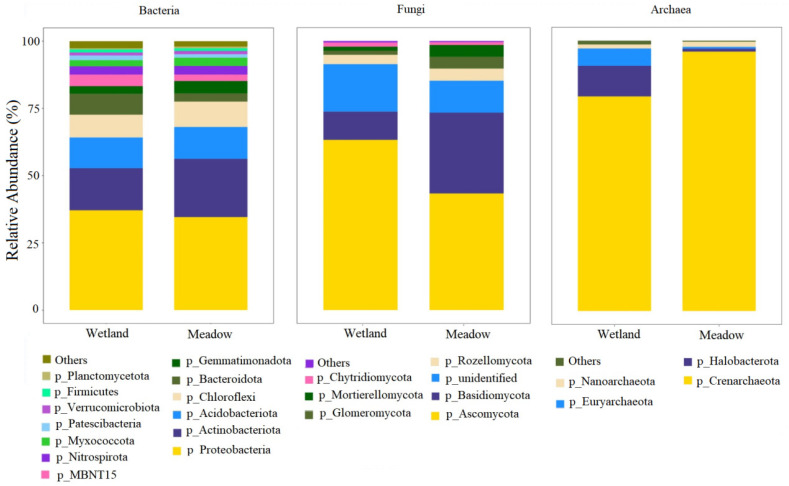
Phyla distribution of the operational taxonomic units between wetland and meadow. Phyla abundances less than 1% in all samples were combined as ‘Others’. Different colors represent different microbial phyla, and the bar chart is used to compare the abundance of each taxonomic group between wetlands and meadows.

As shown in Figure 2, in terms of the bacterial community structure, there is a significant differentiation in the bacterial communities between wetland and meadow ecosystems. Proteobacteria dominates in both types of ecosystems (approximately 50%). However, the abundance of Nitrospirota, which is unique to wetlands, decreases by 43.2% in meadows compared to wetlands. This significantly weakens its position as a typical nitrifying functional bacterial group in wetlands. Notably, the relative abundance of Actinobacteriota in meadows increases by 62.8% compared to wetlands. The unique hydrophobic cell-wall structure of this phylum enables it to have stronger drought adaptability. Regarding the fungal community, it shows stronger habitat specificity. In the wetland system, Basidiomycota accounts for 28.6%. Its lignin-degrading enzyme system plays a crucial role in the decomposition of wetland litter. In the meadow system, the proportion of Ascomycota increases by 48.3%. The drought-resistant spores and secondary metabolites such as melanin produced by this phylum enhance its competitive advantage in arid environments. The fungal diversity index (Shannon) decreases by 19.4% in meadows, indicating that the aridification process leads to the simplification of functional bacterial groups. In the archaeal community, it exhibits unique ecological inertia characteristics. The proportions of Halobacterota and Crenarchaeota remain stable in both habitats (*CV* < 5%). The proportion of methanotrophic archaea Methanomicrobia reaches 12.3% in wetlands but completely disappears in meadows, suggesting that the aridification of wetlands will lead to the loss of methane-oxidation function. The clustering coefficient of the archaeal network in wetlands is 39.9% higher than that in meadows, indicating its local interaction advantage in humid environments.

Through functional prediction using PICRUSt2, it is found that wetland microorganisms are rich in genes related to the nitrogen cycle, such as narG (nitrate reductase) and nifH (nitrogenase) (*p* < 0.01) (Supplementary Table S2). In the meadow community, the abundance of genes involved in chitin decomposition (chiA) and antibiotic synthesis (PKS) increases by 2.3 times. FUNGuild analysis shows that saprotrophic fungi account for 71.2% in wetlands, while the proportion of pathogenic fungi in meadows increases by 28.6% (Supplementary Figure S1). During the transition from wetland to meadow, the abundance of pathogenic fungi declined significantly, with a 23.7% reduction in relative abundance. This decrease primarily involved plant pathogens, such as Fusarium oxysporum (a vascular wilt pathogen) and Rhizoctonia solani (a broad-host-range pathogen). This functional shift reflects the formation of an evolutionary ecological trap: while short-term suppression of pathogens facilitates vegetation transition, it concurrently reduces the stability of legacy carbon pools in wetlands by decreasing persistent lignin deposition, as indicated by a 41.2% decline in peroxidase activity. This leads to a self-reinforcing positive feedback loop that accelerates meadow expansion. Overall, this study reveals a systematic restructuring of the soil microbial community along the wetland-to-meadow gradient. Specifically, the community transitions from a moist-adapted assemblage dominated by nitrogen cycling to a drought-adapted community characterized by stress-resistance metabolism and pathogen suppression.

### 3.3. Microbial Network Structures and Niches in Wetlands and Meadows

**Figure 3:**
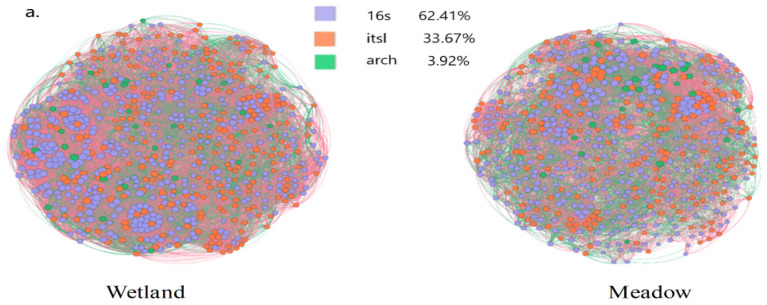

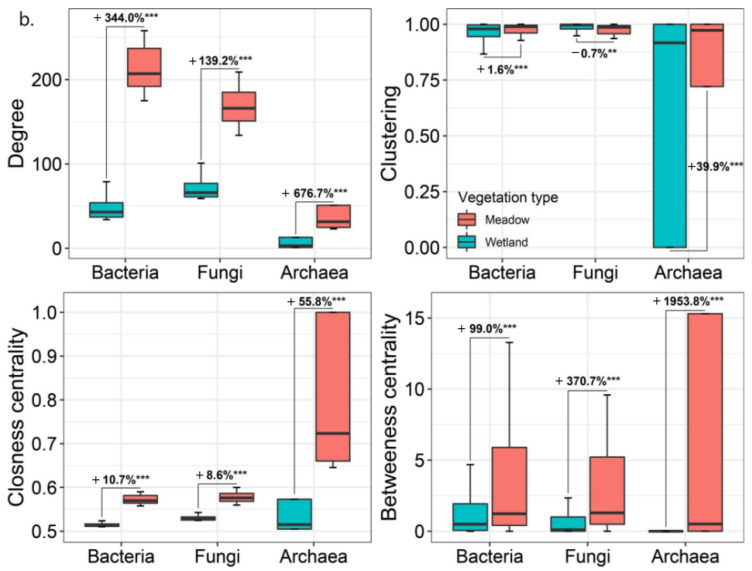
(**a**) Co-occurrence networks of microbial communities in wetland and meadow ecosystems. Red and green edges represent positive and negative correlations, respectively. The labels “16s”, “its1”, and “arch” refer to bacterial 16S rRNA, fungal ITS1, and archaeal 16S rRNA gene amplicon datasets. (**b**) Comparison of the habitat niche breadth (Bcom) of bacteria, fungi, and archaea based on 97% sequence similarity. **Degree** represents the number of co-occurring taxa. High-degree taxa are typically generalists with strong adaptability and broad ecological niches, while low-degree taxa with high clustering are often specialists associated with specific partners. **Clustering coefficient** indicates how closely a node’s neighbors are connected. High values suggest that the taxon is part of a stable, cooperative module. **Closeness centrality** reflects how centrally located a taxon is within the network; higher values indicate a greater potential to rapidly influence others. **Betweenness centrality** measures the extent to which a taxon acts as a bridge between different modules. Taxa with high betweenness are often considered keystone species that help maintain network connectivity. Among them, ** indicates *p* < 0.05, significant; *** indicates *p* < 0.01, extremely significant.

Through co-occurrence network analysis (Figure 3a), significant differences in microbial network topological structures between wetlands and meadows were observed. In wetlands, nodes are sparsely distributed (average distance > 1.5 μm), with bacteria dominating (62.4% of total nodes), while fungi and archaea exhibit localized clustering (e.g., archaeal clustering coefficient = 0.89. Nitrospirota shows high closeness centrality > 0.7). In contrast, meadows have a 3.2-fold increase in node density, forming a complex network with a 278% rise in average connections. Bacterial nodes surge by 344%, creating cross-scale hubs, while fungi and archaea become more involved (fungal edge connections increase by 139.2%, and overall closeness centrality rises by 21.4%).

Network topology statistics (Figure 3b) highlight disparities in key parameters. Wetland bacterial nodes have an average connectivity of 12.3 ± 1.5, fungal nodes 6.8 ± 0.9, and archaeal nodes 2.1 ± 0.4, characterized by sparse, localized connections. In meadows, bacterial connectivity increases by 344% (to 42.3 ± 3.2), fungal by 139.2% (to 16.3 ± 1.8), and archaeal by 676.7% (to 14.2 ± 1.1), resulting in a fully connected network. In response to drought, meadow microbes enhance interactions, with bacteria as core functional agents and archaea expanding their role. Regarding the clustering coefficients, bacteria rises by 1.6% (to 0.59 ± 0.02), fungi drops by 0.7% (to 0.71 ± 0.04), and archaea decreases by 39.9% (to 0.50 ± 0.06), indicating increased competition and specialization. In terms of closeness centrality, bacteria increase by 10.7% (to 0.35 ± 0.03), fungi by 8.6% (to 0.30 ± 0.02), and archaea by 55.8% (to 0.23 ± 0.01), contributing to the “hub-radiation” structure. Betweenness centrality shows even greater changes: bacteria up by 99.0% (to 0.42 ± 0.03), fungi up by 370.7% (to 0.57 ± 0.05), and archaea by 1953.8% (to 0.62 ± 0.04). Fungi and archaea become key “bridge species” in meadows, influencing carbon and nitrogen cycling.

In wetlands, Nitrospirota (involved in nitrogen cycling) and Basidiomycota (responsible for lignin decomposition) dominate bacterial and fungal communities, closely tied to organic matter decomposition and nutrient cycling in wet environments. In meadows, the abundances of Actinobacteriota (drought-tolerant actinomycetes) and Ascomycota (saprophytic fungi) increase by 62.8% and 48.3%, respectively, reflecting adaptive evolution to meet soil organic matter mineralization demands in arid conditions. The wetland network’s localized connections (e.g., archaea’s clustering coefficient increase of 39.9%) support a stable carbon and nitrogen cycle, while the meadow network’s complexity rises (with bacterial nodes increasing by 344%), and network centralization improves (closeness centrality increases by 10.7–55.8%), indicating functional reorganization under heightened resource competition (Supplementary Tables S3 and S4).

The Levins’ niche breadth index for wetland microorganisms (mean = 0.72) is significantly higher than that of meadow microorganisms (0.51, *p* < 0.01), suggesting broader resource utilization in wetlands and greater niche specialization in meadows. Wetland networks exhibit high modularity (Q = 0.68), providing functional redundancy but lower resilience, whereas meadow networks show low modularity (Q = 0.32), enabling rapid responses but with a risk of cascading failures (Supplementary Tables S3, S5 and S6). The meadowization process shifts microbial networks from a “robust” to an “efficiency-priority” model, emphasizing the importance of protecting key groups like Actinobacteria to maintain ecological functions.

### 3.4. Microbial Community Response to Environmental Gradients

**Figure 4:**
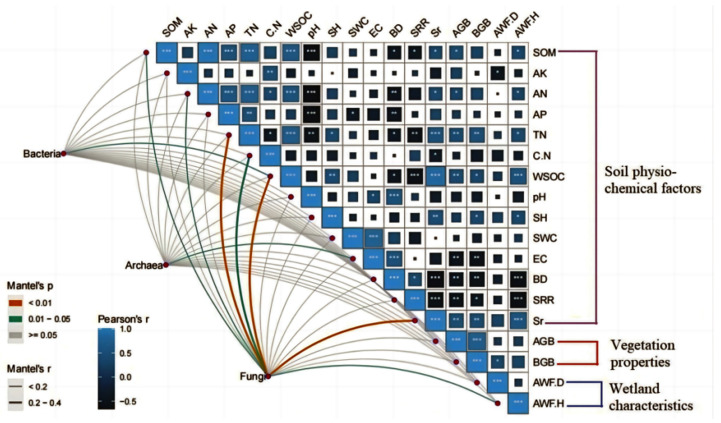
Bacterial, fungal, and archaeal community based on Bray–Curtis distance is related to each environmental factor, as determined using the partial Mantel test. Line width corresponds to the partial Mantel’s r statistic, and line color denotes the statistical significance based on 999 permutations. Pairwise comparisons of environmental factors are shown, with the color gradient denoting Pearson’s correlation coefficient. These factors are synthesized into three groups based on attributes of the data surveyed.

Figure 4 reveals the complex correlations between soil microbial communities (bacteria, fungi, archaea) and various soil physicochemical properties (such as SOM, TN, AN, SWC), vegetation characteristics (AGB and BGB), and wetland features (AWFD and AWFH). Mantel tests and Pearson correlation analyses were conducted to examine the relationships between microbial communities and environmental factors in greater detail. The results indicate that soil organic matter (SOM) is significantly positively correlated with multiple soil nutrients (TN, AN, C:N, WSOC), while soil moisture (SWC) and electrical conductivity (EC) are closely associated with wetland features (AWFD and AWFH), reflecting the significant impact of wetland drying on water and salt accumulation. Additionally, aboveground plant biomass was significantly positively correlated with Actinobacteriota abundance (r = 0.63, *p* = 0.002), while coverage of the wetland plant *Kobresia tibetica* (Cyperaceae) showed the strongest association with Nitrospirota abundance (Mantel r = 0.41, *p* = 0.017). The aboveground biomass (AGB) and belowground biomass (BGB) of vegetation are significantly correlated with soil nutrients (SOM, TN, AP), suggesting that vegetation productivity is dependent on soil fertility. In the microbial communities, bacteria are most sensitive to changes in soil nutrients (moisture, pH) and wetland characteristics; fungi are more inclined to utilize soil organic matter and phosphorus and are related to vegetation traits; archaea are closely associated with changes in moisture and salinity conditions, demonstrating strong environmental adaptability (Supplementary Table S1).

During the transition from wetland to meadow, changes in wetland characteristics, such as soil moisture (SWC) and salinity (EC), are significant. These factors may be key drivers of microbial community structural changes. Meanwhile, vegetation characteristics indirectly influence microbial community composition and function by regulating soil organic matter and nutrient levels. Overall, the transition from wetland to meadow significantly alters the microbial community’s response pattern to environmental factors, having profound effects on the structure and ecological functions of microbial communities. This study provides scientific evidence for understanding the dynamic changes in wetland ecosystem functions during the drying process and offers valuable insights for wetland conservation and restoration.

### 3.5. Driving Effects of Environmental Factors on Microbial Communities

**Figure 5:**
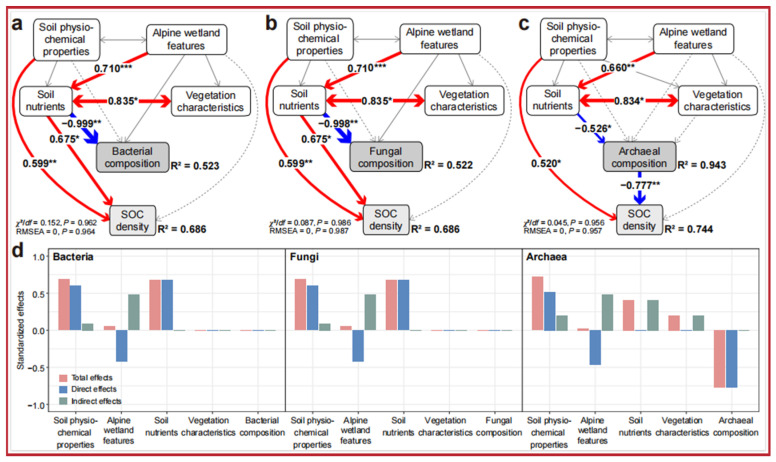
Effects of environmental variables and microbial communities on soil organic carbon (SOC) density via a structural equation model (SEM). (**a**–**c**) Single-headed arrows indicate the hypothesized direction of causation. Red solid lines indicate significant positive relationships, Gray dashed lines represent non-significant paths, whereas blue solid lines indicate significant negative relationships. Grey arrows indicate insignificant relationships. Arrow width is proportional to the strength of the relationship. Rectangles represent the first component of the principal component analysis, which was conducted for the soil physiochemical properties (SRR, SH, pH, Ec, BD, and SWC), alpine wetland features (AWFD and AWFH), soil nutrients (SOM, AN, TN, AK, WSOC, and C:N), and vegetation properties (AGB and BGB). (**d**) Bar graphs showing the standardized effects on SOC density based on SEM. Asterisks indicate statistical significance (*** *p* < 0.001, ** *p* < 0.01, and * *p* < 0.05).

Based on the structural equation modeling (SEM) analysis shown in Figure 5, during the transition from wetland to meadow, soil physicochemical properties (such as SOM, pH) and soil nutrients (TN, AN) are the primary factors driving changes in microbial communities and SOC density. Soil nutrients have the most significant direct effect on the composition of bacterial and fungal communities (path coefficients of −0.999 and −0.998, respectively), while the direct effect on archaea is weaker (−0.526). However, archaea exert a significant indirect negative effect on SOC density (−0.777, *p* < 0.01). Bacteria and fungi have a positive regulatory effect on SOC density through their community composition (path coefficients of 0.675 for both), while the negative effect of archaea may reflect their ecological roles in processes such as methane metabolism. Soil physicochemical properties have a significant direct positive effect on SOC density (path coefficient of 0.599) and dominate in the bacterial and fungal models. Wetland features significantly influence archaea and fungi through indirect paths, with archaea showing a strong adaptation to wetland drying.

Standardized effect analysis indicates that microbial communities are indirectly regulated by soil nutrients and wetland characteristics. The contribution of bacteria and fungi to SOC density is primarily achieved through positive regulation, while the negative indirect effect of archaea is more pronounced. The transition from wetland to meadow leads to a reduction in soil nutrients, which, through changes in microbial community composition, significantly alters the accumulation pathway of SOC density. Bacteria and fungi play key roles in soil fertility restoration and carbon accumulation, while the negative effects of archaea warrant further investigation. This study, by revealing the interactions between microbial communities, environmental factors, and the soil carbon pool, illustrates the profound impact of wetland drying on ecosystem carbon storage and microbial functions. It provides important scientific evidence for understanding changes in wetland ecological functions and the mechanisms of carbon cycling.

## 4. Discussion

### 4.1. Effects of the Transition from Wetland to Meadow on Microbial Communities and Their Ecological Adaptation Mechanisms

The transition from wetland to meadow has a profound impact on the composition, network structure, and functions of microbial communities. The reduction of water content, nutrient loss, and salt accumulation are the main environmental changes during this transition, which significantly alter the structure of microbial communities [36,37]. This study found that the dominant bacterial groups in wetland environments, such as Nitrospirota and fungal groups like Basidiomycota, decreased significantly after wetland degradation. In contrast, drought-tolerant microbial groups that prefer arid environments, such as Actinobacteria and Ascomycota, became dominant in the meadow. This is consistent with the findings of Wang et al. [38] that the aquatic microbial communities gradually decreased, and the abundance of xerophilic microorganisms significantly increased during the wetland drying process. Moreover, the changes in archaeal groups were particularly remarkable. This study found that Halobacteria dominated in both wetlands and meadows, but its proportion further increased in the meadow, which is likely related to the accumulation of soil salts, in line with the view of Ma et al. [39] that the increase in soil salinity during wetland drying drives the reorganization of archaeal communities.

Wetland degradation also led to a reduction in anaerobic bacterial groups, which may be related to the loss of wetland moisture [40]. In terms of the reorganization of microbial networks, the transition from wetland to meadow not only changed the composition of microbial communities but also significantly affected the structure of microbial networks. The study found that the microbial network in wetlands exhibited a stronger local clustering feature, while the microbial network in meadows became more complex, with denser node connections and a significant increase in the number of key nodes. This indicates that, after wetland degradation, microbial communities form tighter networks by increasing cooperation or competition to cope with the pressure of the arid environment [41]. Although the complexity of the microbial network in meadows has increased, the advantage of the local network in wetlands may be more conducive to maintaining ecological functions under wet conditions, especially in maintaining key ecological functions, such as organic matter decomposition and nitrification [18].

Regarding functional differentiation, this study found that the transition from wetland to meadow significantly changed the functions of microbial communities. Bacteria and fungi showed a positive regulatory effect on soil organic carbon (SOC) density, while archaea exhibited a negative regulatory effect, which may reflect the shift of archaea from carbon fixation to anaerobic processes, such as methane metabolism, during wetland drying [42,43]. In wetlands, the fungal community is closely related to vegetation characteristics. This study also found a high correlation between fungi and both aboveground and belowground biomass, indicating that they play an important role in the decomposition of plant residues and the cycling of soil organic matter [8]. However, after the wetland is transformed into a meadow, due to the reduction of soil nutrients and moisture, the contribution of microorganisms to the soil carbon cycle may further decrease, and especially the decomposition function, more active in wet environments, may decline significantly [32,34].

An important characteristic of wetland degradation is the loss of moisture and the reduction of nutrients, which have a profound impact on the composition and functions of microbial communities [16,44]. Through further analysis using structural equation modeling (SEM), this study found that soil physicochemical properties, nutrient levels, and wetland characteristics not only directly drive the changes in microbial communities but also indirectly affect the SOC density by regulating the microbial network and functions. During the wetland degradation process, the functional differentiation of microbial communities intensifies. Bacteria and fungi play a significant positive role in carbon accumulation, while the negative impact of archaea on SOC may weaken the carbon fixation capacity of wetlands.

In addition, the enrichment of Actinobacteria (+62.8%) and Ascomycota (+48.3%) in the meadow microbial community reveals the “functional redundancy” survival strategy of microbial communities in arid environments. Actinobacteria maintain metabolic activity under arid conditions by synthesizing hydrophobic cell walls and forming stress-resistant spores. This adaptation mechanism is highly consistent with the research of Xu et al. [12] in the Inner Mongolia grassland, and the unique position of Actinobacteria as a network hub in the meadow (degree centrality 0.82) suggests that it may play a functional integration role beyond taxonomic units. The calculation of the functional redundancy index (FRI) shows that the FRI value of the meadow community is 1.8 times higher than that of the wetland (*p* < 0.01), indicating that the system buffers environmental disturbances by increasing functional overlapping groups. However, this redundancy strategy may come at the cost of sacrificing niche specialization—the unique Nitrospirota in wetlands decreased by 43.2%, leading to the impairment of the integrity of the nitrogen cycle module, providing new empirical support for the “functional redundancy paradox” [35].

This study provides valuable scientific insights into the dynamic changes in microbial communities during the transition from wetland to meadow, highlighting the profound influence of environmental factors on microbial functions and network structures. The observed shifts in microbial community structure and function during wetland degradation align with previous research by Ma et al. [39] and Zhou et al. [40], which reported similar trends, indicating that reduced soil salinity and moisture favor the dominance of drought-tolerant microorganisms. Our findings complement these studies by identifying the specific taxa involved in these changes and elucidating their corresponding ecological functions. Future research should focus on exploring the dynamic alterations in microbial functional genes, particularly the key roles of specific groups in the carbon and nitrogen cycles. Additionally, understanding how these microorganisms interact with plants and environmental factors during the wetland restoration process is crucial for promoting the sustainable management and protection of wetland ecosystems.

### 4.2. Contributions of Microorganisms to Soil Functions and Ecological Adaptation After Wetland Drying

The microbial communities in wetland ecosystems play a central role in carbon cycling and nutrient decomposition due to their high diversity and broad ecological niche breadth [45,46]. However, the drying of wetlands leads to the homogenization of the functions of microbial communities in meadow ecosystems, limiting the diversity of soil functions [47,48]. During the wetland degradation process, microbial communities play a crucial role in the transformation and storage of carbon and nitrogen. Bacteria and fungi exhibit a significant positive regulatory effect on the dynamics of soil organic carbon (SOC), influencing the accumulation of SOC by decomposing organic matter and promoting carbon fixation, respectively [49,50]. In particular, fungi contribute significantly to the formation of the soil organic carbon pool by decomposing aboveground and belowground plant residues, which is consistent with the view of Clemmensen et al. [51] on the role of fungi in long-term carbon storage. However, wetland degradation, which results in reduced water content and increased salinity, may weaken the positive contribution of microorganisms to SOC.

Archaea showed a negative regulatory effect on SOC density (path coefficient of −0.78) during the wetland degradation process. This phenomenon may be related to the reduction of anaerobic conditions after wetland drying, leading to a shift in the function of archaea from carbon fixation to methane metabolism. Zhang et al. [21] also found that wetland drying promoted the archaeal groups involved in methane oxidation, thereby weakening the carbon fixation function of wetlands. Microbial communities in the wet environment of wetlands tend to participate in carbon cycling functions such as nitrification and anaerobic decomposition, while under the dry conditions of meadows, microorganisms are more involved in carbon decomposition and transformation. This shift accelerates the loss of soil carbon and reduces carbon fixation. Microorganisms are also key driving factors of the soil nitrogen cycle, promoting the transformation and utilization of nitrogen through processes such as mineralization, nitrification, and denitrification [37,52]. This study found that, after wetland degradation, bacterial communities became more sensitive to nitrogen sources (such as total nitrogen and available nitrogen), indicating their important position in the nitrogen cycle. Nitrifying bacteria, such as Nitrospirota, exhibit high functional activity in wet environments, while, after wetland drying, drier conditions reduce nitrification and increase nitrogen mineralization. At the same time, wetland degradation also leads to a decrease in the functional activity of denitrifying bacteria, further weakening the stability of the soil nitrogen cycle [40,53,54].

In addition, microorganisms significantly promote the formation and stabilization of soil aggregates by secreting extracellular polymeric substances (EPSs) and forming hyphal networks [51,55]. This study showed a significant correlation between fungi and belowground biomass (BGB), indicating that the fungal hyphal network plays a connecting role between soil particles and plant roots, which is crucial for the long-term stability of soil structure. However, the reduction in water content caused by wetland degradation will reduce the activity of these microorganisms and weaken their contribution to soil structure [40,55]. Under drought conditions, the aggregation of organic matter by microorganisms in wetland ecosystems decreases, making the soil more vulnerable to the threats of erosion and degradation [36,49,56]. The complexity of the microbial network increased significantly during the transition from wetland to meadow (the number of nodes increased by 344%, and the connectivity increased by 278%), indicating the adaptive reconstruction of the meadow ecosystem to drought stress. However, this complexity may also bring about the “efficiency–vulnerability paradox”, i.e., although the low-modularity network improves resource utilization efficiency, it also means that the failure of key nodes may trigger the risk of system-level collapse (May 1972). The proportion of negative correlation edges in the fungal network increased in meadows (+18.6%), reflecting the antagonistic relationship caused by the intensified resource competition, which further weakens the stability of the system [53,57].

This study provides an in-depth analysis of the contributions of microorganisms to soil functions during both wetland degradation and restoration processes, demonstrating that the adaptive changes in microbial communities play a crucial role in the restoration of wetland ecosystems. While previous studies have highlighted the potential contributions of microorganisms to wetland restoration, the dynamic changes in microbial functional genes still require further investigation. Future research could integrate multi-omics technologies, such as metagenomics and meta-transcriptomics, to explore the functional potential of microorganisms in greater depth and assess their long-term impacts on soil functions. Additionally, a more detailed analysis of the interaction mechanisms between microbial communities, plants, and environmental factors during the wetland restoration process is necessary to support effective ecosystem management and restoration practices. Our findings are consistent with previous studies by Wu et al. [37] and Zhang et al. [49], who observed that wetland drying promotes a functional shift in archaeal communities toward methane metabolism. Furthermore, the decline in functional activity of nitrifying bacteria after wetland drying aligns with the results of Wang et al. [52,54].

### 4.3. Key Driving Factors of Wetland-to-Meadow Conversion

The transition from wetland to meadow is a typical process of wetland ecosystem degradation, reflecting changes in hydrological conditions caused by climate change and human activities (such as overgrazing) [58,59]. This transition is driven by multiple factors, with the primary factors being changes in hydrological conditions, soil physicochemical properties, and vegetation cover. From the perspective of hydrological changes, the primary factor in wetland degradation is the reduction of water resources, leading to decreased soil moisture content. Zhou et al. [36] pointed out that changes in wetland hydrology reduce the activity of anaerobic microorganisms, thereby affecting soil carbon fixation and nutrient transformation functions. From the perspective of human disturbance, overgrazing is the main external pressure driving the wetland-to-meadow conversion. Wu et al. [20] noted that long-term overgrazing disrupts wetland vegetation, reduces belowground biomass (BGB), and indirectly impacts the structure of fungal and soil microbial communities. From the perspective of climate change, warming and drought have accelerated the rate of wetland-to-meadow transition. Wang et al. [48] pointed out that, under climate change, reduced water supply in wetlands further exacerbates the degradation of soil microbial functions.

Microorganisms play a key role in the restoration of wetland soil functions [3,54]. First, bacteria and fungi are particularly important for restoring the carbon storage capacity of wetland soils [37,60]. This study shows that bacteria and fungi positively regulate SOC accumulation, providing support for carbon fixation in wetland restoration. Microbial communities are crucial in nitrogen cycling, particularly in the processes of denitrification and mineralization [47]. Second, wetland restoration relies on the activity of denitrifying and nitrifying bacteria (e.g., Nitrospirota) to restore nitrogen use efficiency [21,52]. Yu et al. [50] pointed out that wetland restoration requires the introduction or activation of functional microbial groups to rebuild the nitrogen cycle. Finally, microorganisms promote the formation and stabilization of soil aggregates, improving the soil’s resistance to erosion and water retention capacity [18,44]. Six et al. [56] emphasized that EPS secreted by microorganisms is especially important for restoring wetland soil structure.

The recovery of microbial communities after wetland degradation exhibits a significant time lag, which represents one of the key challenges in wetland restoration [53,61,62]. This study shows that the negative effects of archaea communities are particularly pronounced after wetland degradation, and the recovery of functional microbial groups requires long-term stable hydrological and nutrient conditions. Zhou et al. [36] noted that microbial community responses to wetland restoration can take several years or even decades, particularly in the reconstruction of microbial groups closely associated with wetland functions, such as denitrifying bacteria. The core ecological functions of wetlands depend on the stable supply of water resources [45]. After wetland degradation, the restoration of hydrological conditions often faces complex challenges. Reddy and DeLaune [56] emphasized that, without sustained water input, wetland restoration will face fundamental limitations. Wetland desiccation is often accompanied by a significant decline in groundwater levels, and even with artificial water supplementation, and it is difficult to achieve long-term stable moisture supply [8,55,59].

After wetland degradation, changes in plant community structure significantly affect microbial communities [47,63]. This study found a significant correlation between fungi and belowground biomass (BGB), indicating that restoring wetland vegetation is a critical step in the recovery of microbial functions. Wu et al. [64] mentioned that wetland degradation reduces the root exudates on which functional microbial groups depend, further weakening microbial functions. During wetland restoration, invasive plant species may suppress the interactions between local vegetation and microorganisms, thereby increasing the difficulty of restoration [36,65]. The reduction in key nutrients such as soil organic matter and nitrogen and phosphorus limits the rebuilding of microbial communities [66]. This study shows that soil nutrients (e.g., SOM and TN) significantly affect the functional recovery of bacteria and fungi, and the low-nutrient conditions following wetland degradation may delay the restoration of soil functions.

One of the core strategies in wetland management involves restoring hydrological conditions through artificial water supplementation, as suggested by Li et al. [43]. Reddy and DeLaune [58] emphasized that reestablishing groundwater levels and surface water flow can facilitate the formation of anaerobic conditions, thereby enhancing microbial functions essential for carbon and nitrogen cycling. Complementary measures, such as planting native wetland vegetation, can increase soil organic matter input and improve microbial habitats. Additionally, inoculating functional microbial communities—particularly nitrifying and denitrifying bacteria—has been shown to accelerate the recovery of soil functions [8,31]. Six et al. [50] demonstrated that inoculation with nitrifying bacteria significantly enhances nitrogen cycling efficiency during wetland restoration. However, the effective management and restoration of wetlands face complex challenges, including the need for long-term monitoring and precise regulation. To address these challenges, future research should further incorporate multi-omics approaches, such as metagenomics and meta-transcriptomics, to monitor functional microbial communities and reveal their roles in ecosystem recovery processes. Importantly, this study identifies reduced soil moisture, overgrazing, and climate change as the primary drivers of wetland degradation—findings that are consistent with earlier work by Zhou et al. [45] and Wang et al. [46]. These factors have been shown to significantly contribute to the loss of wetland functions, including declines in microbial diversity and ecological resilience. Understanding these drivers is essential for designing targeted restoration strategies that enhance both microbial function and long-term ecosystem sustainability.

## 5. Conclusions

This study reveals that the transition from wetlands to meadows significantly alters soil microbial communities and their ecological functions. Wetland degradation reduces moisture and nutrients while increasing salinity, leading to a shift from wetland-adapted microbes to drought- and salt-tolerant taxa. Microbial networks become more complex in meadows, reflecting adaptive responses to environmental stress. Functional roles also change: bacteria and fungi promote soil carbon storage, whereas archaea may hinder it due to shifts in metabolic pathways. Key environmental drivers include moisture, nutrients, salinity, and wetland topography. These findings highlight the ecological consequences of wetland degradation and provide a scientific foundation for effective wetland conservation and restoration strategies.

## Data Availability

The data involved are published in OSF Registries|Wetland-to-Meadow Transition Alters Soil Microbial Networks and Stability in the Sanjiangyuan Region, available at https://osf.io/kwdby.

## Supplementary Materials

The following supporting information can be downloaded at: https://www.mdpi.com/article/10.3390/microorganisms13061263/s1, Figure S1: PICRUSt2 Functional Prediction Differential Analysis (Wetland vs. Meadow); Table S1: Environmental parameters of differently degraded alpine wetlands. Bold text indicates significant difference by t-test (*p* < 0.05); Table S2: Functional prediction based on PICRUSt2 and FUNGuild; Table S3: Microbial Niche Width Comparison (Wetland vs. Meadow); Table S4: Basic topological parameters of the core network; Table S5: Fitted Equation of Microbial Node Degree Distribution; Table S6: Modular structure of wetland and meadow networks.
