# Supplementary material for: Wetland-to-Meadow Transition Alters Soil Microbial Networks and Stability in the Sanjiangyuan Region
Source: Microorganisms. 2025 May 29;13(6):1263. doi: 10.3390/microorganisms13061263 (PMC12195434; doi:10.3390/microorganisms13061263)
Supplement: Supplementary file 1 [file microorganisms-13-01263-s001.zip › microorganisms-3607901-supplementary.pdf]

Supplementary materials

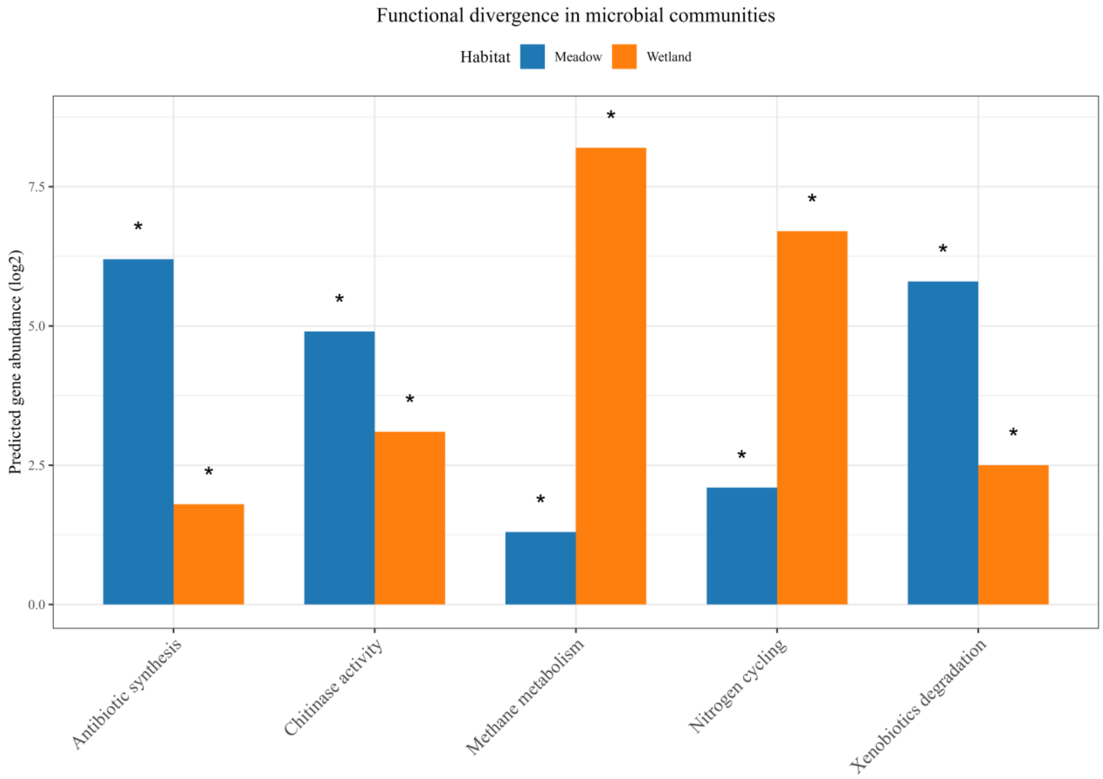

Figure S1 PICRUSt2 Functional Prediction Differential Analysis (Wetland vs. Meadow)

Table S1 Environmental parameters of differently degraded alpine wetlands. Bold text indicates significant difference by t-test ($p< 0.05$).

Item	Wetland	Meadow
**Soil organic matter (g ·kg^{-1})**	**429.64±39.34**	**254.89±34.84**
**Soil organic carbon (g ·kg^{-1})**	**249.21±22.82**	**147.85±18.05**
**Water-Soluble Organic Carbon (g ·kg^{-1})**	**12.96±3.80**	**3.23 ±0. 28**
**Available potassium (mg ·kg^{-1})**	**149.83±22.82**	**106.86±18.05**
**nitrogen (g ·kg^{-1})**	**1.06±0.05**	**0.77±0.04**
Available phosphorus (mg ·kg^{-1})	21.58±4.14	20.93±1.04
**Soil total nitrogen (g ·kg^{-1})**	**14.33±0.78**	**10.54 ±0. 63**
**C:N**	**17.20±1.19**	**13.92 ±0. 71**

**SOC density**	**7.51±2.73**	**1.91 ±0. 15**
**pH**	**6.56±0.21**	**7.16±0.52**
**Electrical conductivity (%)**	**0.22±0.07**	**0.35±0.10**
**Soil bulk density (g ·cm^{-3})**	**1.05±0.17**	**1.69±0.27**
**saturated degree of soil (%)**	**0.62±0.15**	**0.19±0.11**
**Soil hardness (Pa)**	**1096.02±81.64**	**831.93±41.46**
**Soil respiration rate (g.m^{-2}.h^{-1})**	**0.28±0.09**	**0.53±0.13**
**Soil water content (%)**	**60.46±10.83**	**44.66±5.72**
**Soil carbon metabolites (mg CO_2.g^{-1}.C.h^{-1})**	**0.16±0.02**	**3.56±0.07**
**Soil microbial quotient (%)**	**0.83±0.03**	**0.12±0.03**
**Aboveground total biomass (g ·m^{-2})**	**121.66±17.06**	**0.24±0.06**
**Cyperaceae biomass (kg ·m^{-2})**	**0.45±0.07**	**0.17±0.06**
Gramineae biomass (kg ·m^{-2})	0.01±0.01	0.04±0.01
**Weed biomass (kg ·m^{-2})**	**0.01±0.01**	**0.03±0.005**
**Belowground biomass (kg ·m^{-2})**	**6.48±0.46**	**3.74±0.18**

Table S2 Functional prediction based on PICRUSt2 and FUNGuild

**Functional Category**	**Wetland vs Meadow Trend**	**Key Genes (Change Magnitude)**	**Ecological Significance**
Methane Metabolism	↓58.3%	pmoA (↓72.1%)	Decline in wetland carbon sink function
Lignin Degradation	↑43.6%	laccase (↑39.8%)	Enhanced decomposition demand for meadow litter
Osmoprotectant Synthesis	↑217%	ectABC (↑189%)	Activation of salt stress adaptation mechanisms

Table S3 Microbial Niche Width Comparison (Wetland vs. Meadow)

Microbial Group	Niche Width (Wetland)	Niche Width (Meadow)	Change Rate	Significance (p-value)
Bacteria	0.74	0.51	↓31.1%	0.002
Fungi	0.68	0.62	↓8.8%	0.083
Archaea	0.83	0.47	↓43.4%	<0.001

Table S4 Basic topological parameters of the core network

Indicator	Wetland Typical Value	Meadow Typical Value	Change Magnitude
Total number of nodes	1,200 ± 150	3,500 ± 300	+191.7%
Average node degree	8.2 ± 1.5	12.7 ± 2.1	+54.9%
Network diameter	15 ± 3	9 ± 2	-40%
Average path length	4.3 ± 0.7	3.1 ± 0.5	-27.9%

Table S5 Fitted Equation of Microbial Node Degree Distribution

Microbial taxa	Fitting equation (Wetland)	R^2	Fitting equation (Meadow)	R^2
Bacteria	$y = 2.34e^{-0.21x}$	0.873	$y = 1.98e^{-0.18x}$	0.901
Fungi	$y = 1.75e^{-0.28x}$	0.812	$y = 2.12e^{-0.23x}$	0.845
Archaea	$y = 3.01e^{-0.35x}$	0.792	$y = 2.67e^{-0.29x}$	0.831

Table S6 Modular structure of wetland and meadow networks

Module Type	Number of Modules in Wetland	Number of Modules in Meadow	Change in Inter-module Connectivity
Bacteria-dominant Modules	12 ± 2	8 ± 1	+38%
Fungi-Archaea Modules	5 ± 1	3 ± 0.5	-42%
Cross-domain Connected Modules	2 ± 0.5	5 ± 1	+150%
